# Particle Morphometrics of Glycopyrronium-Formoterol-Budesonide Inhalation Blends Influence Pulmonary Deposition and Bronchodilator Response in Chronic Obstructive Pulmonary Disease (COPD): A Gamma Scintigraphy Study

**DOI:** 10.7759/cureus.116954

**Published:** 2026-09-26

**Authors:** Gaurav K Jain, Abha Mishra, Nilesh Chandra, Manu Madan

**Affiliations:** 1 DSIR-DPSRU-CRTDH (Department of Scientific and Industrial Research–Delhi Pharmaceutical Sciences and Research University–Common Research and Technology Development Hub), Center for Advanced Formulation Technology, Department of Pharmaceutics, DPSRU, New Delhi, IND; 2 Department of Physiology, Jodhpur Institute of Engineering and Technology (JIET) Medical College and Hospital, Jodhpur, IND; 3 Division of Discovery Research, Indian Council of Medical Research, New Delhi, IND; 4 Department of Respiratory and Sleep Medicine, Medanta Hospital, Noida, IND; 5 Department of Pulmonology, Max Smart Super Speciality Hospital, Saket, New Delhi, IND

**Keywords:** aerosol particle size, copd, dry powder inhaler, gamma scintigraphy, metered-dose inhaler, peripheral airway deposition

## Abstract

Background: The therapeutic efficacy of inhaled triple-combination therapy in chronic obstructive pulmonary disease (COPD) depends on efficient delivery to peripheral airways. This study evaluated the influence of particle morphometrics on pulmonary deposition and bronchodilator response to glycopyrronium-formoterol-budesonide (GFB) inhalation formulations.

Methods: Three commercial GFB formulations (X, Y, and Z) delivered via metered-dose inhaler (MDI) and dry powder inhaler (DPI) were characterized using dynamic light scattering and scanning electron microscopy. In a randomized, open-label pilot study, twenty-four patients with mild-to-moderate COPD inhaled technetium-99m (99mTc) radiolabeled formulations. Regional lung deposition was assessed by planar gamma scintigraphy, and central-to-peripheral (C/P) ratios were calculated. Forced expiratory volume in one second (FEV1) was measured at baseline and 30 minutes post-inhalation.

Results: Significant inter-brand differences in particle morphometrics were observed. Brand X demonstrated a narrow particle distribution (1.3±0.26 µm) with uniform spherical morphology, while brands Y and Z showed relatively broader, polydisperse distributions with irregular particles. MDIs (MIX, MIY, MIZ) consistently outperformed DPIs (DIX, DIY, DIZ), producing greater total lung deposition (23.4% vs 13.7%; P < 0.001) and improved peripheral deposition. Among MDIs, MIX achieved higher intrathoracic deposition (28.8 ± 2.2% vs 19.0-22.5%; P < 0.001) and peripheral deposition (15.8 ± 3.0% vs 9.8-11.5%; P = 0.012), with a lower C/P ratio (0.82 vs 0.96-1.0). Greater spirometric improvement correlated with enhanced deposition, with the MIX producing the highest FEV1 increase (12.8%; P < 0.05).

Conclusions: The findings suggest that particle size distribution, polydispersity, and surface morphology may influence pulmonary deposition and acute spirometric response in COPD. A relatively narrow respirable particle distribution may support more consistent peripheral airway deposition and short-term spirometric response.

## Introduction

Chronic obstructive pulmonary disease (COPD) represents a major global health challenge, characterised by persistent airflow limitation, chronic airway inflammation, and progressive structural remodelling involving both central and peripheral airways [1,2]. Inhaled pharmacotherapy constitutes the cornerstone of COPD management, enabling targeted drug delivery to the respiratory tract while limiting systemic exposure [3]. Triple-combination regimens comprising a long-acting muscarinic antagonist, a long-acting β₂-agonist, and an inhaled corticosteroid may provide additional clinical benefit over selected dual regimens, particularly in patients with persistent symptoms and/or an increased risk of exacerbations and features associated with a greater likelihood of inhaled corticosteroid (ICS) responsiveness [4,5].

The clinical effectiveness of inhaled therapies depends substantially on the extent and regional distribution of pulmonary drug deposition. In COPD, pathological involvement of the small airways (<2 mm in diameter) is a prominent feature of the disease and contributes importantly to airflow obstruction and disease progression [6,7]. Consequently, achieving adequate drug delivery to the peripheral lung and small airways represents an important therapeutic consideration, particularly for inhaled therapies intended to act throughout the respiratory tract.

Aerosol deposition within the respiratory tract is governed by the complex interplay of formulation attributes, inhaler-device characteristics, and patient-related factors [8]. Among formulation attributes, particle size distribution, aerodynamic diameter, morphology, density, and interparticle interactions can substantially influence aerosolization and regional pulmonary deposition [9,10]. Particles with an aerodynamic diameter of approximately 1-5 µm are generally considered suitable for deposition in the lower respiratory tract [11]. Larger, relatively dense particles are more likely to undergo inertial impaction in the oropharyngeal region and conducting airways, whereas very small particles may remain suspended within the inspired air and be exhaled before effective deposition [12]. Importantly, aerodynamic diameter alone does not fully determine deposition behaviour, as particle density, shape, surface architecture, aggregation state, and electrostatic properties can also influence aerosol transport and deposition.

Beyond particle size, particle morphology and surface architecture, including shape, surface roughness, porosity, density, and aggregation characteristics, can influence powder flow, deagglomeration, aerosolization, and the fine-particle fraction available for pulmonary delivery [9,13,14]. Importantly, the relationship between morphology and aerosol performance is not universally linear and does not depend on a single optimal particle shape. Particle engineering strategies involving porous, corrugated, or appropriately textured surfaces can alter effective density and interparticle interactions and may enhance powder dispersibility and respirable delivery [15]. For example, large, low-density porous particles have shown efficient deep-lung delivery despite having geometric diameters greater than those conventionally associated with pulmonary deposition [15]. Similarly, modifying carrier-particle surface roughness can alter drug-carrier adhesion and detachment, thereby influencing DPI aerosolization performance [13,14]. Thus, particle morphometrics, rather than geometric shape alone, represent potentially important determinants of aerosol performance and pulmonary deposition.

Metered-dose inhalers (MDIs) and dry powder inhalers (DPIs) are widely used platforms for pulmonary drug delivery, each with distinct device- and formulation-dependent characteristics [16]. DPIs rely on patient-generated inspiratory airflow to disperse and deagglomerate the powder, making their performance dependent on device resistance, formulation properties, and the inspiratory manoeuvre. This consideration is particularly relevant in COPD, where inspiratory flow capacity may be reduced in patients with advanced airflow limitation [17,18]. In contrast, pressurized MDIs use propellant-driven aerosol generation and generally require substantially less inspiratory flow than DPIs, although appropriate inhalation technique and coordination between actuation and inspiration remain important for optimal drug delivery [19]. Reported in vivo lung deposition also varies considerably between inhaler platforms, with substantial overlap between devices and considerable dependence on formulation, device design, inhalation technique, and patient characteristics [17].

Despite containing the same active pharmaceutical ingredients, commercially available inhalation products may differ substantially in particle morphometrics because of differences in manufacturing processes, micronization conditions, formulation composition, and excipient characteristics [20]. Such differences may influence aerosolization behaviour and, consequently, the amount and regional distribution of drug reaching the lungs. Although in vitro cascade-impaction studies provide important measures of aerodynamic performance, including emitted dose and fine-particle fraction, these parameters do not fully capture the complexity of in vivo pulmonary deposition, thereby supporting the use of direct imaging techniques for clinical assessment [21].

Planar gamma scintigraphy using technetium-99m (99mTc)-labelled formulations is an established method for quantifying total and regional pulmonary deposition of inhaled products in humans [22,23]. It enables assessment of whole-lung deposition and regional distribution using predefined regions of interest. Measures such as the central-to-peripheral (C/P) ratio or related outer-to-inner deposition indices commonly characterize the relative distribution of aerosol between central and peripheral lung regions [23].

Despite the widespread clinical use of triple-combination inhalers, clinical investigations directly linking particle morphometric characteristics with in vivo pulmonary deposition remain limited. The present study was therefore designed to (i) comprehensively characterize the particle morphometrics of three marketed glycopyrronium-formoterol-budesonide (GFB) formulations; (ii) quantify regional pulmonary deposition using gamma scintigraphy following DPI and MDI administration in patients with mild-to-moderate COPD; and (iii) explore the relationship between particle morphometric attributes, regional pulmonary deposition, and short-term spirometric response.

## Materials and methods

Materials

Three MDIs of GFB containing 9/4.8/160 µg per actuation, along with three corresponding DPIs containing 25/12/400 µg per capsule, coded as X, Y, and Z, were procured from a retail pharmacy in Delhi, India. The MDIs were coded as MIX, MIY, and MIZ, and the DPIs were coded as DIX, DIY, and DIZ. The products were coded to maintain scientific objectivity, neutrality, and confidentiality. Hydrofluoroalkane (HFA) propellant-only MDI canisters and empty aluminium canisters were provided by Glenmark Pharmaceuticals Ltd (Mumbai, India). The radiotracer 99mTc was obtained from the Board of Radiation and Isotope Technology (BRIT, Mumbai, India), and the radioactive experiments were performed at City Imaging Centre (New Delhi, India) under the supervision of a qualified nuclear medicine specialist.

Morphometric characterization of the inhaler blends

Hydrodynamic particle diameterT was determined by dynamic light scattering (DLS; Litesizer 501, Anton Paar, Austria) at 25°C using a 90° detection angle. For MDI samples, a single actuation was discharged into 3.0 mL of deionized water; for DPI samples, capsule contents were dispersed in 3.0 mL of deionized water prior to measurement.

Surface morphology was examined by scanning electron microscopy (SEM; Alpha, SEC Co. Ltd., Gyeonggi-do, Korea) at an accelerating voltage of 10 kV. Samples were mounted on aluminium stubs using conductive carbon adhesive. DPI powders were used directly, whereas MDI aerosols were actuated onto the adhesive surface from a fixed distance. Images were acquired using the instrument's integrated software.

Radiolabeling of inhalers

The marketed MDIs and DPI powder blends were radiolabeled with 99mTc using previously reported indirect and direct radiolabeling methods, respectively [24,25]. For the MDI suspensions, 99mTc was incorporated into the formulation, allowing the radionuclide to associate with the suspended aerosol particles [24]. For the DPI formulations, the blend particles were directly radiolabeled with 99mTc according to the previously described procedure [25]. Uniformity of radiolabel distribution was confirmed by assaying multiple actuations (MDI) or capsule aliquots (DPI); the coefficient of variation was < 1% across replicates. The drug dose in a single puff from a radiolabeled and a non-radiolabeled inhaler did not differ significantly (p>0.05), indicating no change in inhalational delivery following radiolabeling. 

Study design

This single-center, open-label, six-arm, exploratory pilot study was conducted at City Imaging Centre (New Delhi, India) in accordance with the Declaration of Helsinki and the International Council for Harmonisation of Technical Requirements for Pharmaceuticals for Human Use - Good Clinical Practice (ICH-GCP) guidelines. The study protocol was approved by the Institutional Ethics Committee, Good Society for Ethical Research, Delhi (Approval No. GSER/2025/BMR-AP/237; dated October 31, 2025). Twenty-four participants were included and randomly allocated to six treatment arms, with four participants assigned to each formulation/device combination (MIX, MIY, MIZ, DIX, DIY, and DIZ). The sample size was selected a priori for this pilot scintigraphic investigation primarily based on feasibility, including participant recruitment, radiolabeled inhaler administration, image acquisition, and regional deposition assessment. The study was intended to generate preliminary estimates of deposition and inter-subject variability and to provide information for planning a subsequent adequately powered confirmatory study. Accordingly, the study was not designed or powered to establish definitive differences in pulmonary deposition or clinical efficacy between formulations. The limited sample size and exploratory design were therefore considered when interpreting all between-group comparisons.

Study population

Twenty-four adults, aged 18-60 years, with physician-diagnosed mild-to-moderate COPD, defined by a post-bronchodilator forced expiratory volume in one second (FEV1) of 60-80% [26], and those receiving one or more inhaled maintenance therapies, were enrolled in the study. Participants were randomly allocated to one of six study groups (n = 4): group 1 - MIX, group 2 - MIY, group 3 - MIZ, group 4 - DIX, group 5 - DIY, and group 6 - DIZ. Participants were enrolled after obtaining written informed consent, including consent for scintigraphic imaging. Demographic and clinical characteristics relevant to pulmonary aerosol deposition, including age, sex, smoking status, body mass index, COPD severity, baseline FEV1, and baseline peak inspiratory flow rate (PIFR), were recorded at screening. Participants with COPD exacerbation and/or any upper/lower respiratory tract infection and those with hypersensitivity to study drugs or a history of cardiovascular disease or an immunocompromised status were excluded.

Study procedures

On the day preceding the study day, participants underwent screening assessments and received standardized training in correct inhaler technique using hydrofluoroalkane (HFA)-only MDI and lactose-filled DPI capsules. For MDI administration, participants were instructed to exhale comfortably to functional residual capacity, position the inhaler appropriately, initiate a slow and deep inhalation simultaneously with actuation, continue inhalation to total lung capacity, and then hold their breath for up to 10 seconds before exhaling. For DPI administration, participants were instructed to exhale away from the device, place the mouthpiece correctly, and perform a rapid and deep inhalation through the device to facilitate powder deaggregation and aerosol generation, followed by a breath-hold of up to 10 seconds. The inhalation maneuvers were demonstrated and supervised by trained personnel. The same standardized instructions were used for all participants to minimize variability due to inhalation technique.

Participants were also trained to use the spirometer. PIFR was assessed during screening and prior to DPI administration. A PIFR of >30 L/min was considered acceptable. Rescue bronchodilators were withheld for at least six hours prior to dosing. On study days, participants inhaled two actuations of radiolabeled MDI or one capsule of radiolabeled DPI. A breath-hold of up to 10 seconds followed each inhalation. After completing inhalation, participants rinsed their mouths with 20 mL of water (expectorated into a shielded container) and washed their faces. Scintigraphic imaging and FEV1 measurements were performed at baseline and 30 min post-inhalation.

Scintigraphic imaging and spirometry

Planar gamma scintigraphy was performed using a dual-head SPECT-CT system (Symbia TruePoint, Siemens, Germany). Posterior thoracic images and a lateral head-and-neck image were acquired within five minutes of inhalation. To quantify extrathoracic and intrathoracic radioactivity, regions of interest (ROIs) were delineated on the acquired images using Syngo processing software (Symbia TruePoint, Siemens, Germany). The extrathoracic ROI encompassed the oral and pharyngeal regions, whereas the intrathoracic ROI encompassed both lungs. To assess regional pulmonary deposition, the intrathoracic lung field was further divided into central and peripheral compartments using the predefined ROI methodology applied consistently across all study images. The central region represented the relatively proximal/central lung area, whereas the peripheral region represented the outer/distal lung area. The same ROI delineation approach was applied to all participants and treatment groups to minimize measurement variability. Radioactivity counts obtained from each ROI were corrected for background activity and physical decay. Regional deposition was expressed as a percentage of the total measured emitted radioactivity. The central-to-peripheral (C/P) ratio was calculated as the ratio of central to peripheral lung counts.

Spirometry was performed using a calibrated digital spirometer (Sonmol, B-Arm Medical Tech, India), and FEV1 was recorded as the highest value from three acceptable efforts. Participants were observed for 4 hours post-dose and discharged without adverse events.

Study endpoints

The primary endpoint was regional airway deposition expressed as a percentage of the total emitted dose. Secondary endpoints included C/P ratio, change in FEV1 from baseline, and incidence of adverse events.

Statistical analysis

Data are presented as mean ± standard deviation (SD). Between-group comparisons were performed using ANOVA with Tukey's post hoc test for multiple comparisons. Device comparisons (MDI vs DPI) were analyzed using paired t-tests. Statistical analyses were conducted using GraphPad Prism, version 8.0 (GraphPad Software, LLC, Boston, MA, USA); P < 0.05 was considered statistically significant.

## Results

Particle size and morphology

SEM revealed marked inter-brand differences in particle morphology and size distribution among both MDI and DPI formulations (Figure 1). Among the MDI formulations, MIX (Figure 1A) exhibited predominantly spherical to near-spherical particles with smooth surfaces and relatively uniform morphology. MIY (Figure 1B) showed comparatively larger particles with irregular shapes and broader size heterogeneity. MIZ (Figure 1C) exhibited aggregated structures with highly irregular particle morphology, suggesting greater heterogeneity than MIX and MIY.

**Figure 1:**
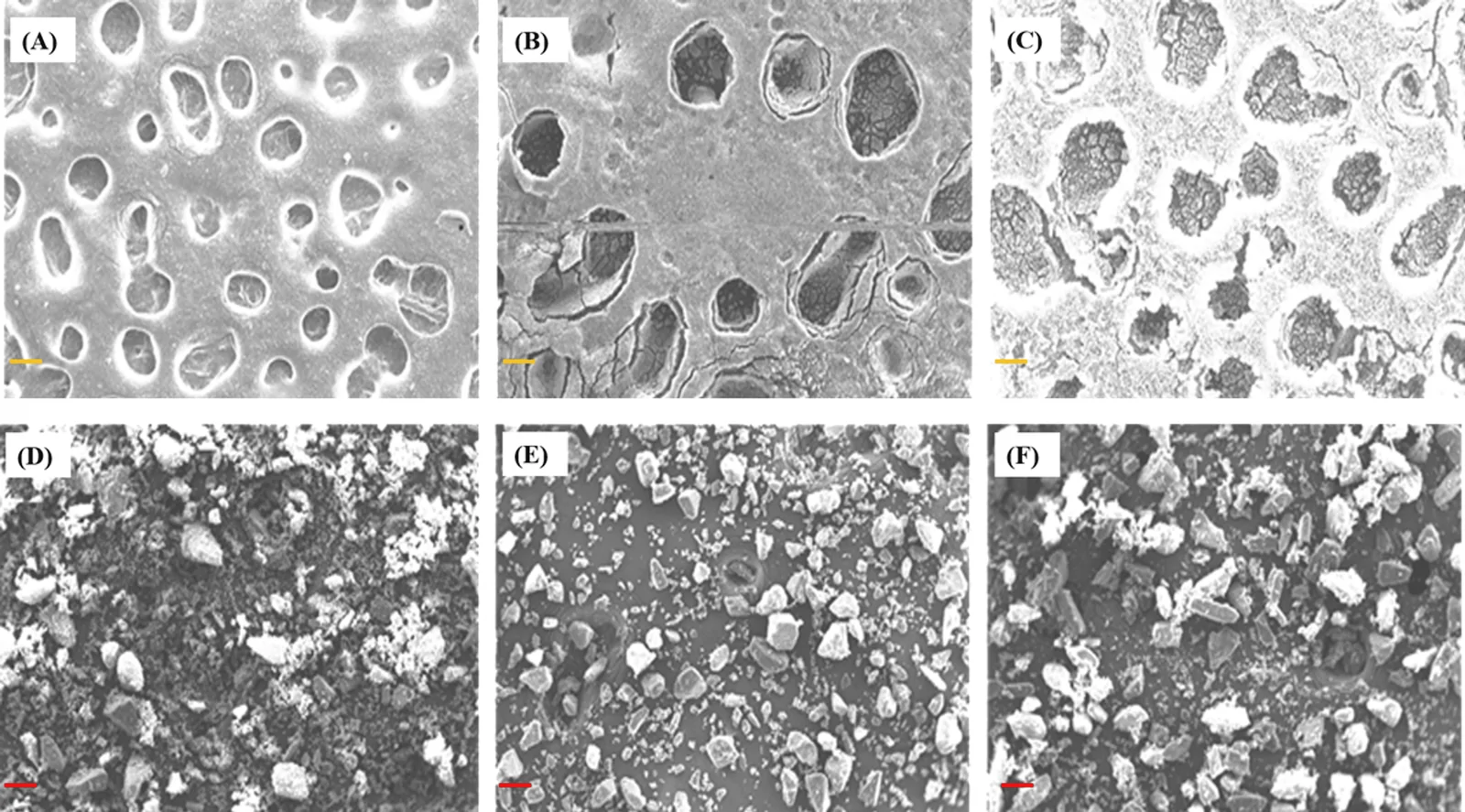
SEM images of blends (A) MIX, (B) MIY, (C) MIZ, (D) DIX, (E) DIY, (F) DIZ. The yellow and red bars represent scales of 1.0 µm (×200) and 10.0 µm (×200), respectively. MIX, MIY, and MIZ are codes for metered-dose inhalers (MDIs), and DIX, DIY, and DIZ are codes for dry powder inhalers (DPIs). SEM, scanning electron microscopy.

Among the DPI formulations, notable differences were also observed in blend characteristics. DIX (Figure 1D) contained a dense population of fine particles distributed around larger carrier particles, indicating a relatively high fraction of micronized drug particles. In contrast, DIY (Figure 1E) and DIZ (Figure 1F) showed coarser, more heterogeneous blends with larger crystalline carrier and fewer fine particles. In particular, DIZ exhibited pronounced irregular crystalline structures across a broader size range.

DLS analysis supported the SEM observations and confirmed significant differences in hydrodynamic size (HD) and polydispersity (PDI) among formulations (Figure 2). For MDI formulations, MIX showed the smallest mean hydrodynamic diameter (1.2 µm) with the lowest polydispersity index (0.25), indicating a relatively narrow and uniform size distribution. MIY and MIZ exhibited larger mean particle sizes of 4.1 µm and 5.2 µm, respectively, with higher PDIs (0.39 and 0.38), reflecting broader, more heterogeneous distributions.

**Figure 2:**
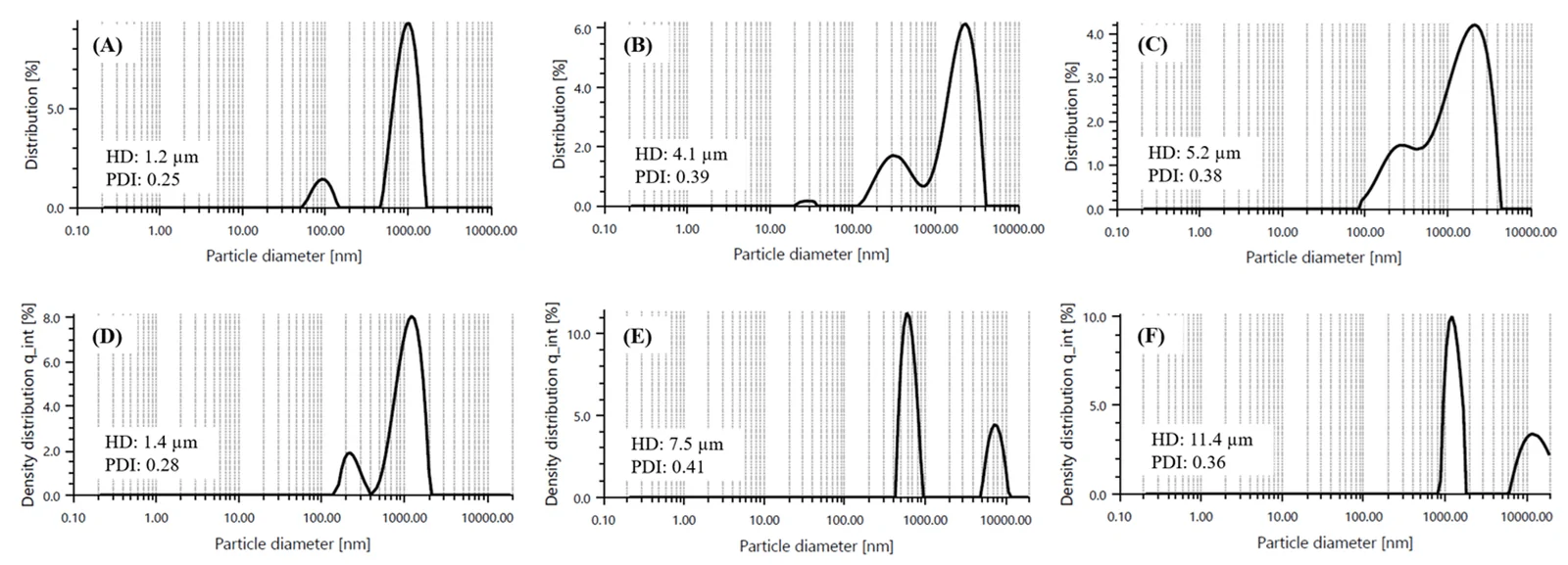
Particle size of blends (A) MIX, (B) MIY, (C) MIZ, (D) DIX, (E) DIY, (F) DIZ. MIX, MIY, and MIZ are codes for metered-dose inhalers (MDIs), and DIX, DIY, and DIZ are codes for dry powder inhalers (DPIs).

Similarly, among DPI formulations, DIX demonstrated the smallest mean hydrodynamic diameter (1.4 µm) with a relatively narrow distribution (0.28), consistent with its high fine-particle fraction observed by SEM. In contrast, DIY and DIZ showed substantially larger mean particle sizes (7.5 µm and 11.4 µm, respectively) and higher PDIs (0.41 and 0.36), indicating greater heterogeneity and aggregation. Overall, Brand X formulations (MIX and DIX) exhibited smaller particle sizes and narrower distributions than Brands Y and Z, suggesting potentially superior aerosolization and deeper pulmonary deposition.

Regional pulmonary deposition

A total of 24 participants were enrolled in the study. All participants were male and non-smokers. Mean age and BMI did not vary significantly among the study groups (p > 0.05). Similarly, baseline FEV1 values did not differ significantly across cohorts (p > 0.05). PIFR varied slightly among the DPI cohorts.The DIY group had the highest mean PIFR; however, this difference was not statistically significant compared with the DIX and DIZ groups (p > 0.05).

Quantitative gamma-scintigraphic analysis demonstrated differences in the regional distribution of inhaled aerosol among the three MDI formulations (MIX, MIY, and MIZ) and three DPI formulations (DIX, DIY, and DIZ). Table 1 presents subject-level deposition data and corresponding mean ± SD values, and Figure 3 shows representative scintigraphic images from one participant for each formulation.

**Table 1:** Regional airway deposition of MDIs and DPIs (%) by gamma scintigraphy. Peak inspiratory flow (PIF) rate > 30 L/min; Central deposition: upper and larger airways; Peripheral deposition: distal airways and alveolar regions; ^a^ P < 0.05 and ^b^ P > 0.05 compared to other brands. The missing fraction or percent corresponds to stomach deposition, mouthwashout, and device retention. MIX, MIY, and MIZ are codes for metered-dose inhalers (MDIs), and DIX, DIY, and DIZ are codes for dry powder inhalers (DPIs).

Inhalers	MDIs			DPIs		
Brand	MIX	MIY	MIZ	DIX	DIY	DIZ
Extrathoracic						
Subject 1	48.0	53.0	53.0	38.0	34.0	38.0
Subject 2	46.0	48.0	58.0	39.0	48.0	47.0
Subject 3	43.0	56.0	59.0	33.0	34.0	43.0
Subject 4	42.0	48.0	57.0	32.0	45.0	47.0
Mean±SD	44.8±2.8^b^	51.3±3.9	56.8±2.6	35.5±3.5^a^	40.3±7.3	43.8±4.3
Intrathoracic						
Subject 1	32.0	23.0	18.0	16.0	12.0	8.0
Subject 2	28.0	24.0	21.0	20.0	14.0	12.0
Subject 3	27.0	22.0	20.0	16.0	18.0	10.0
Subject 4	28.0	21.0	17.0	13.0	10.0	15.0
Mean±SD	28.8±2.2^b^	22.5±1.3	19±1.8	16.3±2.9^a^	13.5±3.4	11.35±3.0
Central						
Subject 1	12.0	10.0	11.0	8.0	7.0	6.0
Subject 2	15.0	13.0	9.0	9.0	8.0	5.0
Subject 3	12.0	10.0	11.0	10.0	12.0	6.0
Subject 4	13.0	11.0	8.0	11.0	7.0	8.0
Mean±SD	13.0±1.4^b^	11.0±1.4	9.8±1.5	9.5±1.3^a^	8.5±2.4	6.3±1.3
Peripheral						
Subject 1	20.0	13.0	7.0	8.0	5.0	2.0
Subject 2	13.0	11.0	12.0	11.0	6.0	7.0
Subject 3	15.0	12.0	9.0	6.0	6.0	4.0
Subject 4	15.0	10.0	11.0	2.0	3.0	7.0
Mean±SD	15.8±3.0^b^	11.5±1.3	9.8±2.2	6.8±3.8^a^	5.0±1.4	5.0±2.4

**Figure 3:**
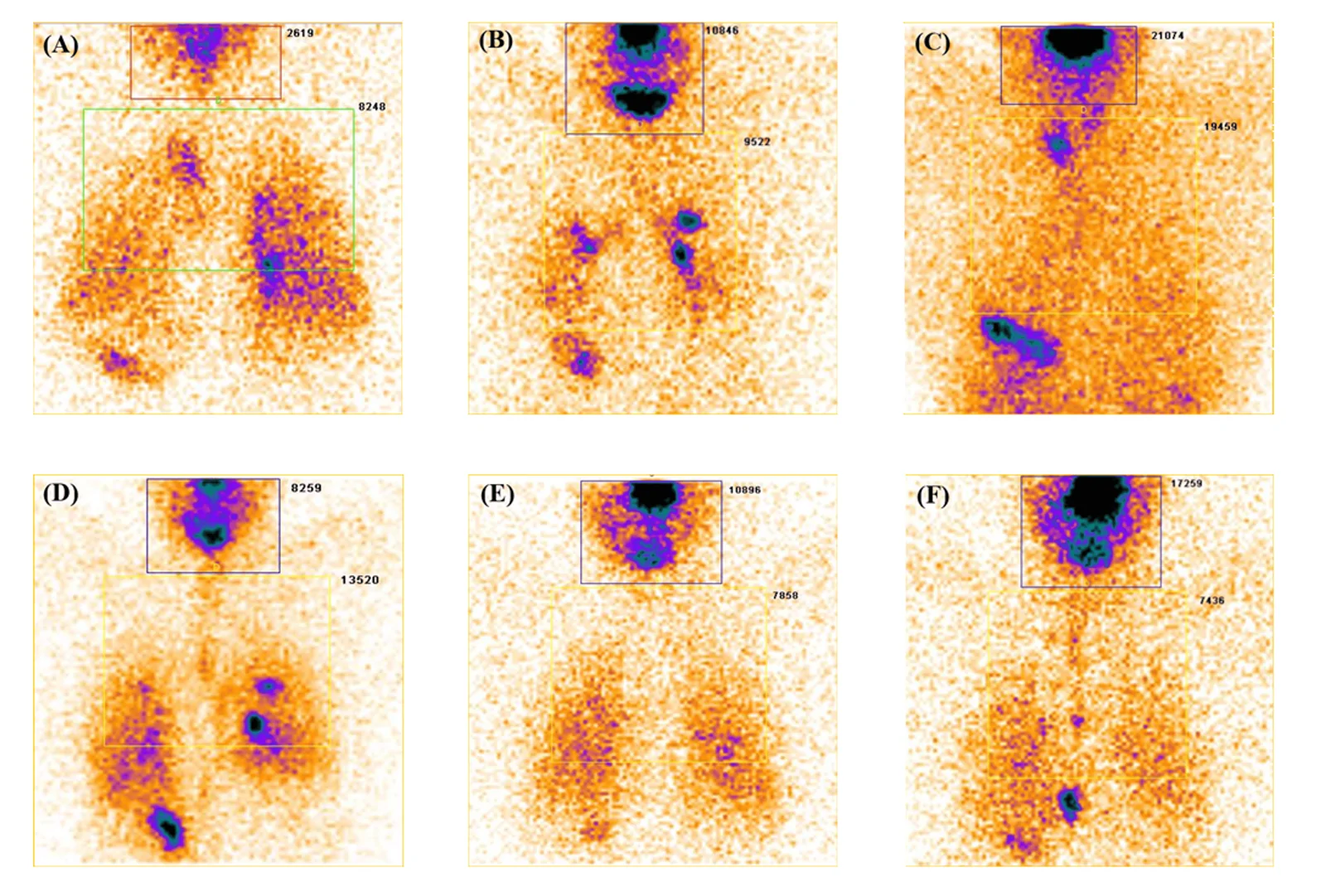
Scintigraphy images showing regional airway deposition of one participant from each group (A) MIX, (B) MIY, (C) MIZ, (D) DIX, (E) DIY, (F) DIZ. MIX, MIY, and MIZ are codes for metered-dose inhalers (MDIs), and DIX, DIY, and DIZ are codes for dry powder inhalers (DPIs).

Device comparison

Across all three brands, MDI formulations showed higher intrathoracic deposition compared with their DPI counterparts. Mean intrathoracic deposition for DPIs ranged from 11.3-16.3%, whereas MDIs achieved substantially higher deposition values of 19.0-28.8%. This device-related difference was statistically significant (P = 0.0001), indicating improved pulmonary delivery efficiency with MDIs.

Regional deposition analysis further revealed greater central-lung deposition with MDIs than with DPIs. Mean central deposition ranged from 9.8-13.0% for MDIs versus 6.3-9.5% for DPIs (P = 0.03). Importantly, MDIs also achieved higher peripheral lung deposition, with mean values ranging from 9.8-15.8%, compared with 5.0-6.8% for DPIs (P = 0.012). Correspondingly, C/P ratios were lower for MDI formulations (range 0.82-1.0) than for DPI formulations (range 1.4-1.8), indicating superior distal airway penetration with MDI delivery (Figure 4).

**Figure 4:**
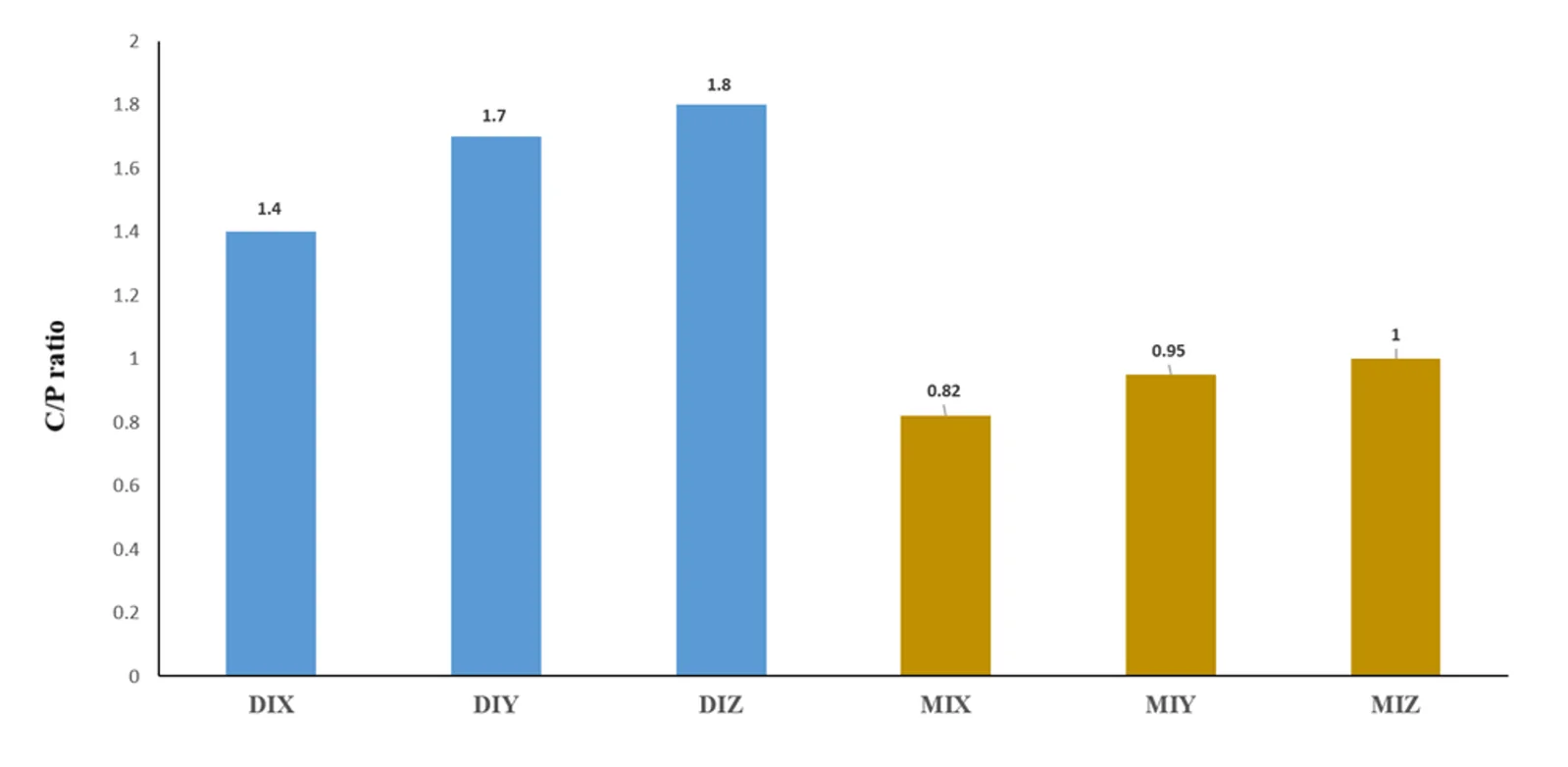
Central-to-peripheral (C/P) deposition ratio of various inhalation formulations.

Extrathoracic (oropharyngeal) deposition was higher with MDI (range 44.8-56.8%) than DPI (range 35.5-43.8%; P = 0.0015), reflecting the higher initial aerosol velocity and lack of blend retention by propellant-driven systems.

Inter-brand comparison

Among MDIs, brand X (MIX) achieved the highest intrathoracic deposition (28.8 ± 2.2%), followed by MIY (22.5 ± 1.3%) and MIZ (19.0 ± 1.8%). Inter-brand differences were statistically significant (P < 0.001), indicating substantial variability in pulmonary delivery performance among MDI formulations.

A similar trend was observed for peripheral lung deposition, with MIX achieving the highest deposition (15.8 ± 3.0%), followed by MIY (11.5 ± 1.3%) and MIZ (9.8 ± 2.2%) (P = 0.012). The C/P ratio was lowest for MIX (0.82), indicating the most favourable distal airway targeting.

In contrast, inter-brand differences among DPI formulations were less pronounced and did not reach statistical significance. Mean intrathoracic deposition ranged from 11.3 ± 3.0% to 16.3 ± 2.9%, with DIX showing the highest deposition (16.3 ± 2.9%), followed by DIY (13.5 ± 3.4%) and DIZ (11.3 ± 3.0%) (P = 0.127). These findings suggest comparatively lower, less differentiated pulmonary deposition profiles among DPI formulations than among MDIs.

Spirometric response

Baseline and post-inhalation FEV1 mean values are summarized in Table 2. At baseline, mean FEV1 values were comparable across all three brands, irrespective of inhaler type, indicating no clinically meaningful differences in pre-dose lung function among the treatment arms.

**Table 2:** Baseline and post-inhalation mean FEV1 values ^a ^P < 0.05 vs. baseline; MIX, MIY, and MIZ are codes for metered-dose inhalers (MDIs), and DIX, DIY, and DIZ are codes for dry powder inhalers (DPIs). FEV1, forced expiratory volume in one second.

Group	Mean FEV1 (SD)	
	Baseline	Post-inhalation
MIX	71.0 (5.89)	83.8 (6.08)^a^
MIY	71.2 (6.13)	80.0 (3.45)^a^
MIZ	70.3 (7.37)	78.8 (5.74)^a^
DIX	70.5 (3.70)	78.3 (7.23)^a^
DIY	71.5 (4.65)	75.3 (6.75)
DIZ	70.2 (3.77)	74.8 (5.62)

All treatments improved FEV1 at 30 minutes post-inhalation (Table 2). The magnitude of bronchodilatory response was consistently greater with MDI formulations compared with their corresponding DPI formulations across all brands. Among all, MIX produced the greatest improvement in FEV1, with a mean increase of 12.8 percentage points from baseline (P < 0.05), followed by MIY and MIZ. Among DPI formulations, DIX showed a significant improvement, whereas DIY and DIZ produced comparatively smaller, statistically non-significant changes. Notably, the rank order of spirometric improvement closely paralleled the scintigraphic pulmonary deposition profile, with formulations achieving higher lung deposition demonstrating greater bronchodilatory efficacy.

Safety

No serious adverse events were reported. Transient mild oropharyngeal discomfort was noted in two participants following MDI administration; symptoms resolved spontaneously without intervention.

## Discussion

This gamma scintigraphy study provides preliminary in vivo evidence of an association between particle morphometric characteristics, regional pulmonary deposition, and short-term spirometric response in patients with COPD. The findings establish a link between optimized particle engineering and improved targeting of the peripheral airways, with implications for formulation development and therapeutic optimization.

Particle morphometrics as determinants of regional deposition

The observed association between particle morphometric characteristics and scintigraphic deposition patterns suggests that particle properties may influence aerosol behaviour within the respiratory tract. In this pilot study, Brand X, characterized by a relatively narrow size distribution, smaller particle size (1.3 ± 0.26 µm), and comparatively uniform morphology, tended to show greater peripheral lung deposition and lower C/P ratios across the inhalers evaluated at usual tidal inspiratory flow rates. These findings should, however, be interpreted cautiously, as regional deposition may also be influenced by patient-specific factors, inhalation characteristics, and inter-subject variability. Particle size is an important determinant of aerosol deposition. Particles larger than approximately 5 µm are more susceptible to inertial impaction and may preferentially deposit in the oropharyngeal and central airway regions, whereas gravitational sedimentation and Brownian diffusion become relatively more important as particle size decreases, potentially facilitating penetration into the peripheral airways and alveolar regions [11,12].

The relatively small and more uniform particles observed for Brand X may reduce the proportion of larger particles susceptible to proximal airway impaction while potentially favoring retention within the respirable size range. These characteristics may partly explain the deposition pattern observed in this study. However, the relationship between particle size and regional deposition is complex and is also dependent on aerodynamic diameter, inspiratory flow, airway geometry, and patient-specific respiratory characteristics.

Beyond particle size, morphological characteristics may also influence aerodynamic behaviour [14,15]. The relatively spherical and smooth-surfaced particles observed in Brand X may exhibit more consistent aerodynamic characteristics than particles with irregular or heterogeneous morphology. Such differences could potentially contribute to variations in regional deposition; however, the present study does not establish a direct causal relationship between particle morphology and pulmonary deposition.

In comparison, the more irregular and heterogeneous particle populations observed for Brands Y and Z may be associated with greater variability in aerodynamic behaviour. Differences in particle shape and surface characteristics could influence particle transport and deposition within the respiratory tract, although these effects may vary between individuals and with inhalation conditions [11,14].

Polydispersity represents another particle characteristic that may influence aerosol deposition [27]. The comparatively low PDI observed for Brand X suggests a more homogeneous particle population, whereas the higher PDI profiles observed for Brands Y and Z indicate greater particle-size heterogeneity. These differences may broaden the distribution of aerodynamic behaviors, potentially affecting the relative proportions of particles depositing in the oropharyngeal, central, and peripheral lung regions [27]. Nevertheless, this pilot study remains exploratory regarding the contribution of polydispersity to the observed scintigraphic deposition patterns, which warrants confirmation in larger, controlled studies.

Device-dependent modulation of particle morphometric effects

The generally higher deposition observed with MDI delivery across the formulations evaluated in this pilot study suggests a potential interaction between particle morphometric characteristics and device-specific aerosolization mechanisms. MDIs showed greater intrathoracic and peripheral deposition than DPIs, with correspondingly lower C/P ratios, which may indicate a tendency toward greater distal airway deposition. However, these observations should be interpreted cautiously, particularly given the potential influence of inter-subject variability and other device- and patient-related factors.

DPI performance is influenced by the patient's inspiratory flow, which helps overcome interparticulate cohesive forces and facilitates powder deaggregation [17]. In patients with COPD, variations in peak inspiratory flow rate (PIFR) and inspiratory capacity may affect powder dispersion and deaggregation, potentially resulting in the emission of particle agglomerates with aerodynamic characteristics different from those of the primary particles [18]. This mechanism may partly influence the extent to which differences in primary particle morphometry are reflected in regional pulmonary deposition and could contribute to the relatively smaller inter-brand differences observed among the DPI formulations in the present study. In contrast, MDI aerosolization is less dependent on patient-generated inspiratory flow for initial particle dispersion because aerosol generation is driven by the propellant system [19]. The relatively high initial aerosol velocity associated with MDIs may contribute to increased oropharyngeal deposition, as observed in the present study. Nevertheless, a proportion of the aerosol was also observed to reach the intrathoracic and peripheral lung regions. These findings suggest that the interaction between particle characteristics and device aerosolization behaviour may influence the resulting regional deposition pattern. Nonetheless, the evaluated MDI and DPI products differed in device design, formulation characteristics, nominal dose, and aerosol-generation mechanism; the observed MDI-DPI differences cannot be interpreted as evidence that the device platform alone caused the difference in deposition

Overall, the present observations indicate that particle morphometric characteristics may have different effects depending on the inhaler platform and its aerosolization mechanism. This may have implications for formulation-device co-development, as particle engineering strategies developed for MDI systems may not necessarily translate directly to DPI platforms, where powder deaggregation and interactions with carrier particles can influence aerosol performance [19]. These considerations remain exploratory and would benefit from confirmation in larger studies using controlled inhalation conditions and standardized device-performance assessments. It is also noteworthy that the lung deposition observed for the MDIs evaluated in the present study was numerically lower than that reported for Breztri Aerosphere (budesonide/glycopyrrolate/formoterol fumarate) [28]. However, direct comparison should be made with caution because differences in scintigraphic methodology, classification of extrathoracic and intrathoracic deposition, device design, aerosol characteristics, and the use or absence of a spacer may influence reported deposition values. In addition, subject-related anatomical and physiological characteristics including total lung capacity, vital capacity, airway geometry, and inspiratory flow patterns may contribute to variability in regional pulmonary deposition [29]. Therefore, the observed difference should not be interpreted as evidence of an intrinsic difference in formulation performance without further controlled comparative evaluation.

Morphometric-functional correlations

The observed concordance between particle morphometric characteristics, scintigraphic deposition patterns, and spirometric outcomes suggests a possible relationship between regional pulmonary deposition and functional response. In this pilot study, Brand X, which exhibited smaller, more uniform particle characteristics, showed greater peripheral deposition and a numerically greater improvement in FEV1 when delivered via MDI. Although these findings are biologically plausible, the observed association should be interpreted cautiously, as pulmonary function may be influenced by multiple patient-, disease-, and device-related factors, as well as inter-subject variability.

Small-airway inflammation and remodeling are recognized features of COPD and may contribute to airflow limitation [7]. Therefore, improved delivery of inhaled therapies to the peripheral lung may be relevant to therapeutic response. Previous investigations have reported that aerosols with smaller aerodynamic diameters may be associated with improvements in parameters related to airway conductance, air trapping, and exercise capacity compared with formulations producing predominantly larger-particle deposition [11]. However, the magnitude and clinical relevance of these effects may vary according to disease characteristics, inhalation technique, and treatment formulation.

The ICS component of triple therapy may also potentially benefit from deposition in the smaller airways. Some evidence suggests that small-airway inflammation in COPD may respond to local corticosteroid exposure, although the relationship between regional corticosteroid deposition and clinical responsiveness remains incompletely established [5]. Accordingly, formulations with particle characteristics that favour more distal deposition could potentially influence the pulmonary distribution of the ICS component while also contributing to bronchodilator delivery across different airway regions.

Taken together, the findings of this pilot study provide preliminary evidence of an association between particle morphometric characteristics, regional pulmonary deposition, and spirometric response in triple-combination therapy. They raise the possibility that particle characteristics may represent one of several modifiable formulation factors influencing pulmonary drug distribution and, potentially, therapeutic response. However, the present findings are hypothesis-generating and do not establish a causal relationship between peripheral deposition and clinical efficacy. Larger, adequately powered, and preferably controlled studies are required to determine whether differences in particle morphometry and regional deposition translate into clinically meaningful improvements in COPD outcomes.

Implications for particle engineering strategies

The findings of the present pilot study may have implications for developing and optimizing inhaled formulations. The relatively higher peripheral deposition observed with Brand X, characterized by a narrower particle-size distribution, smaller particle size within the 1-2 µm range, and comparatively uniform, near-spherical morphology, suggests that these characteristics may be associated with differences in regional pulmonary delivery and functional response. However, the findings should be interpreted as exploratory rather than as evidence that these specific particle attributes necessarily result in superior clinical performance. Contemporary particle-engineering approaches provide opportunities to deliberately modulate particle size, morphology, and surface characteristics to optimize pulmonary delivery [30]. The present observations suggest that formulation-development strategies aimed at achieving relatively narrow, uniform particle-size distributions, lower polydispersity, and controlled particle morphology could influence peripheral lung deposition. Nevertheless, optimal particle characteristics likely depend on the drug, formulation composition, inhaler platform, and patient inhalation profile. Further systematic studies are therefore warranted to establish the relative contribution of individual morphometric attributes and to determine whether optimization of these characteristics can translate into consistent improvements in pulmonary deposition and clinically meaningful outcomes.

Methodological considerations and limitations

Gamma scintigraphy provides direct visualization and quantification of regional pulmonary deposition and remains the reference method for assessing in vivo aerosol distribution. Integrating complementary particle characterization techniques (DLS and SEM) with scintigraphic imaging is a methodological strength, enabling correlation of physicochemical attributes with in vivo performance. Several limitations warrant consideration. Firstly, the pilot design and limited sample size preclude definitive conclusions regarding clinical efficacy. Larger studies incorporating repeated dosing and extended follow-up are required to confirm these findings. Secondly, radiolabeling was performed on the blend rather than on the individual drugs, and the differential behaviour of labelled and unlabeled constituents cannot be entirely excluded, although validation studies confirmed that dose metering was preserved. Thirdly, particle characterization was performed using DLS and SEM, which provide complementary yet distinct morphometric information. However, aerodynamic particle sizing via cascade impaction would provide additional characterization relevant to respiratory deposition. The non-equivalent nature of the commercial formulations is an additional limitation. The MDI and DPI products differed not only in device platform but also in nominal dose, formulation composition, and aerosol-generation mechanism. Consequently, the observed differences in deposition cannot be attributed exclusively to particle size, morphology, or polydispersity. Patient-related factors, including inspiratory flow, inhalation maneuver, baseline pulmonary function, and airway geometry, may also have contributed to the observed deposition patterns. Given the small sample size, the study was not designed to independently quantify these contributions. Therefore, the observed relationships between particle morphometrics, pulmonary deposition, and FEV1 should be interpreted as associations rather than causal effects. Lastly, FEV1 represents a global measure of airflow that may incompletely capture regional bronchodilator effects. Future studies incorporating impulse oscillometry, nitrogen washout, or functional imaging techniques may provide enhanced sensitivity to peripheral airway responses and strengthen morphometric-functional correlations.

Clinical perspectives

The substantial inter-brand variability in particle morphometrics and deposition observed in this study raises important clinical considerations. Current bioequivalence frameworks for orally inhaled products rely predominantly on in vitro aerodynamic testing supplemented by pharmacokinetic assessments; however, the relationship between in vitro performance metrics and in vivo regional deposition is imperfect.

The present findings suggest that comprehensive particle morphometric characterization, supplemented by scintigraphic studies, may provide valuable evidence during product development, particularly for formulations intended to target peripheral airways. Such data could support evidence-based device selection.

From a clinical perspective, the data support individualized therapeutic approaches accounting for both formulation particle characteristics and patient inspiratory capacity. Patients with severely reduced PIFR may benefit more from MDI delivery of formulations with optimized particle morphometrics, whereas those with preserved inspiratory function may achieve satisfactory outcomes across device platforms.

## Conclusions

This pilot gamma scintigraphy study provides preliminary in vivo evidence of an association between particle morphometric characteristics, regional pulmonary deposition, and short-term spirometric response in patients with COPD. Formulations with smaller, more uniform particles showed greater peripheral deposition in this study; however, these observations cannot establish an independent causal effect of particle morphometrics because formulation composition, device characteristics, nominal dose, inspiratory flow, inhalation technique, and patient-specific respiratory factors also influence pulmonary deposition. The small sample size, non-equivalent commercial formulations, single-centre open-label design, and short-term assessment further limit the strength and generalizability of the findings. Larger, adequately powered, controlled studies incorporating standardized inhalation conditions and detailed patient-level assessments are warranted to determine the independent contribution of particle characteristics to pulmonary deposition and clinical response.
